# Establishment and Finite Element Analysis of a Three‐dimensional Dynamic Model of Upper Cervical Spine Instability

**DOI:** 10.1111/os.12474

**Published:** 2019-06-26

**Authors:** Xiao‐dong Wang, Min‐shan Feng, Yong‐cheng Hu

**Affiliations:** ^1^ Graduate Department Tianjin University of Traditional Chinese Medicine Tianjin China; ^2^ Spine Department Wangjing Hospital Beijing China; ^3^ Department of Orthopaedic Oncology Tianjin Hospital Tianjin China

**Keywords:** Upper cervical spine, Instability, Dynamic finite element, Biomechanics

## Abstract

**Objectives:**

To establish a dynamic three‐dimensional (3D) model of upper cervical spine instability and to analyze its biomechanical characteristics.

**Methods:**

A 3D geometrical model was established after CT scanning of the upper cervical spine specimen. The ligament of the specimen was fatigued to establish the upper cervical spine‐instability model. A 100‐N preloaded stress was applied to the upper surface of the occipital bone, and then a 1.5‐Nm moment was applied in the occipital‐sagittal direction to simulate upper cervical spine flexion and extension. Subsequently, the 3D dynamic model was established based on trajectory data that were measured using a motion‐capture system. The stress on the main ligament and the relative motion angle of the joint were analyzed.

**Results:**

The shape of the model grid was regular and the total number of its units was 627 000. After finite‐element analysis was conducted, results of the ligament stress and relative movement angle were obtained. After the upper cervical spine instability, the pressure of the alar ligament during the upper cervical spine extension was increased from 2.85 to 8.12 MPa. The pressure of the flavum ligament was increased during the upper‐cervical spine flexion, from 0.90 to 1.21 MPa. The pressure of the odontoid ligament was reduced during the upper cervical spine flexion and extension, from 10.46 to 6.67 MPa and 25.66 to 16.35 MPa, respectively. The pressure of the anterior longitudinal ligament and cruciate ligament was increased to a certain degree during upper cervical spine flexion and extension. The pressure of the anterior longitudinal ligament was increased during flexion and extension, from 7.70 to 10.10 MPa and 10.45 to 13.75 MPa, respectively. The pressure of the cruciate ligament was increased during flexion and extension, from 2.29 to 4.34 MPa and 2.32 to 4.40 MPa, respectively. In addition, after upper cervical spine instability, the articular‐surface relative‐movement angle of the atlanto‐occipital joint and atlanto‐axial joint had also changed. During upper cervical spine flexion, the angle of the atlanto‐occipital joint was increased from 3.49° to 5.51°, and the angle of the atlanto‐axial joint was increased from 8.84° to 13.70°. During upper cervical spine extension, the angle of the atlanto‐occipital joint was increased from 11.16° to 12.96°, and the angle of the atlanto‐axial joint was increased from 14.20° to 17.20°. Therefore, the movement angle of the atlanto‐axial joint was most obvious after induction of instability.

**Conclusion:**

The 3D dynamic finite‐element model of the upper cervical spine can be used to analyze and summarize the relationship between the change of ligament stress and the degree of instability in cervical instability. Frequent or prolonged flexion activities are more likely to lead to instability of the upper cervical spine.

## Introduction

Chronic strains have gradually increased the number of patients with instability of the upper cervical spine. Upper cervical spine instability is a common clinical‐spine disease and an increasing number of doctors are paying attention to it because it can cause a series of clinical symptoms, such as dizziness and nausea^1^, ^2^. However, the location and degree of upper cervical spine instability will have different effects on the clinical manifestations and diagnosis of the disease. Upper cervical spine instability occurs during movement, resulting in difficulty in making accurate diagnoses; biomechanical research may be able to help improve such diagnostic measures. However, due to the lack of financial support for basic research, the biomechanical mechanisms of upper cervical spine instability have not been clarified, which adversely affects progress in corresponding clinical research and in the efficacy of evaluations of this disease. Finite‐element methods have been increasingly used in the field of biomechanics. These methods can visually express the stress inside structures of the human body. In addition, these methods have high reuse value and save on costs; there is no safety risk and the individual differences can be greatly avoided^3^. Hence, finite‐element methodology may greatly ameliorate the shortcomings of studies investigating upper cervical spine instability.

Currently, the main shortcoming of studies on upper cervical spine instability is related to the diagnosis of the disease. Most doctors have a clear consensus on the diagnosis of cervical instability. X‐ray imaging has shown that an angle of more than 11° between the vertebral bodies or a horizontal displacement of more than 3.5 mm between the vertebral bodies can indicate the existence of cervical instability^4^. However, due to the particularity of the anatomical structure of the upper cervical spine, this standard cannot be fully applied. Therefore, difficulties in imaging‐based diagnoses of upper cervical spine instability have been raised^5^.

The dynamic positioning of X‐ray imaging of cervical spine flexion and extension can provide a good reference for the upper cervical spine, and it can also indicate that upper cervical spine instability occurs in the movement process of the cervical spine^6^. However, the currently popular medical‐imaging methods cannot accurately capture and describe the overall process and dynamic characteristics of upper cervical spine instability^7^.

Biomechanical factors are among the most important factors in the development of cervical spondylosis and have important significance in scientific research. The implementation of finite element analysis on upper cervical spine specimens should employ a finite element model with the following characteristics. Finite element models are primarily established *via* geometric modeling, three‐dimensional (3D) coordinate‐instrument modeling, and image modeling. Image modeling is presently a commonly used modeling method in clinical biomechanical research^8^. Generally, CT and MRI data of specimens have been collected in advance and have been generated into a compatible file format and then input into finite‐element‐modeling software. This approach has been used to establish a model of upper cervical spine instability, in which simulations of different working conditions are carried out by finite‐element software. This has enabled comparative analyses of various experimental hypotheses. It can be seen that, compared with traditional simple physical experiments, animal experiments, and *in vitro* specimen experiments, the finite‐element technique can not only reflect the physiological or pathological characteristics of the upper cervical spine more realistically, but it can also improve the accuracy of the results analyses^9^. The application of finite element technology in the field of biomechanics has developed rapidly. Foreign scholars have taken the lead in establishing a 3D finite element model of the lumbar spine and in simulating biomechanical analyses. Domestic research has gradually developed from the establishment of an independent vertebral‐body model to a whole‐spine model, and from the establishment of the finite element model of the spine to research on the basic pathogenesis, preoperative planning, and postoperative evaluation of spinal diseases. However, there have been few studies on the dynamic finite element model of the upper cervical spine established at home and abroad, and no investigations have been conducted on the dynamic model of instability. The high‐quality dynamic finite element model of upper cervical spine instability may better reflect biomechanical changes in the instability of the upper cervical spine, provide a reliable theoretical basis for the imaging diagnosis of the disease, and have great value for scientific research^10^.

Through the study of *in vitro* cervical specimens, the method of a continuous load to induce ligament stress in cervical instability models was established. This approach has been accomplished using motion‐capture technology dynamic measurements of cervical spine physiology, and by inducing the instability of two states of activities. More recently, finite element analysis has been adopted to analyze the mechanics of limited movement in the upper cervical‐spine‐instability model, and to reveal relationships between mechanical changes and instability of the cervical spine ligament^11^.

Finally, dynamic virtual‐interactive technology has been used to establish a dynamic 3D‐simulation model of upper cervical spine instability^12^. The finite element method has been used to analyze the biomechanical characteristics of the upper cervical spine, which has been helpful for the early diagnosis of cervical spine disease and the development of treatment plans, and in the evaluation of treatment efficacy.

## Materials and Methods

### *Experimental Equipment*

The following experimental equipment was used:

1. CT scanner (Siemens SOMATOM Definition dual source CT, Germany; material: rare earth ceramic; high voltage generator: 80 kV; scan time: 0.32 s; slice thickness: 0.25 mm)
2. Motion‐capture system (Vicon T20s, UK; aperture setting: 2.3; frequency: 500 Hz’ distinguishability: 1600 × 1280; pixel: 200 W)
3. Dynamic fatigue‐testing machine (Bose ELF 3200, USA; maximum accelerated speed: 200 G; tensile load: ± 3000 N; torsional load: ±49 Nm; displacement stroke: 50 mm; frequency: 0.00001–100 Hz).

### *Analytical Software*

The following analytical software was used:

1. Medical‐imaging control‐system software Mimics v10.0 (Materialise, Belgium); basic modules: image import, image segmentation, image visualization, image registration, and image measurement.
2. Reverse‐processing software Solid works v2014 (Dassault Systèmes, United States); network requirements: Microsoft's Windows Networking or Active Directory network; Excel and Word: 2003, 2007, 2010, 2013, 2016; server: Windows Server 2003 or above.
3. Sub‐network software Hypermesh v13.0 (Altair, United States); unit quality parameters: aspect <5:1, chord dev, interior angles, jacobian >0.7, skew.
4. Finite‐element‐analysis software Abaqus v6.13 (Dassault Systèmes, United States).

### *Experimental Specimens*

**Figure 1:**
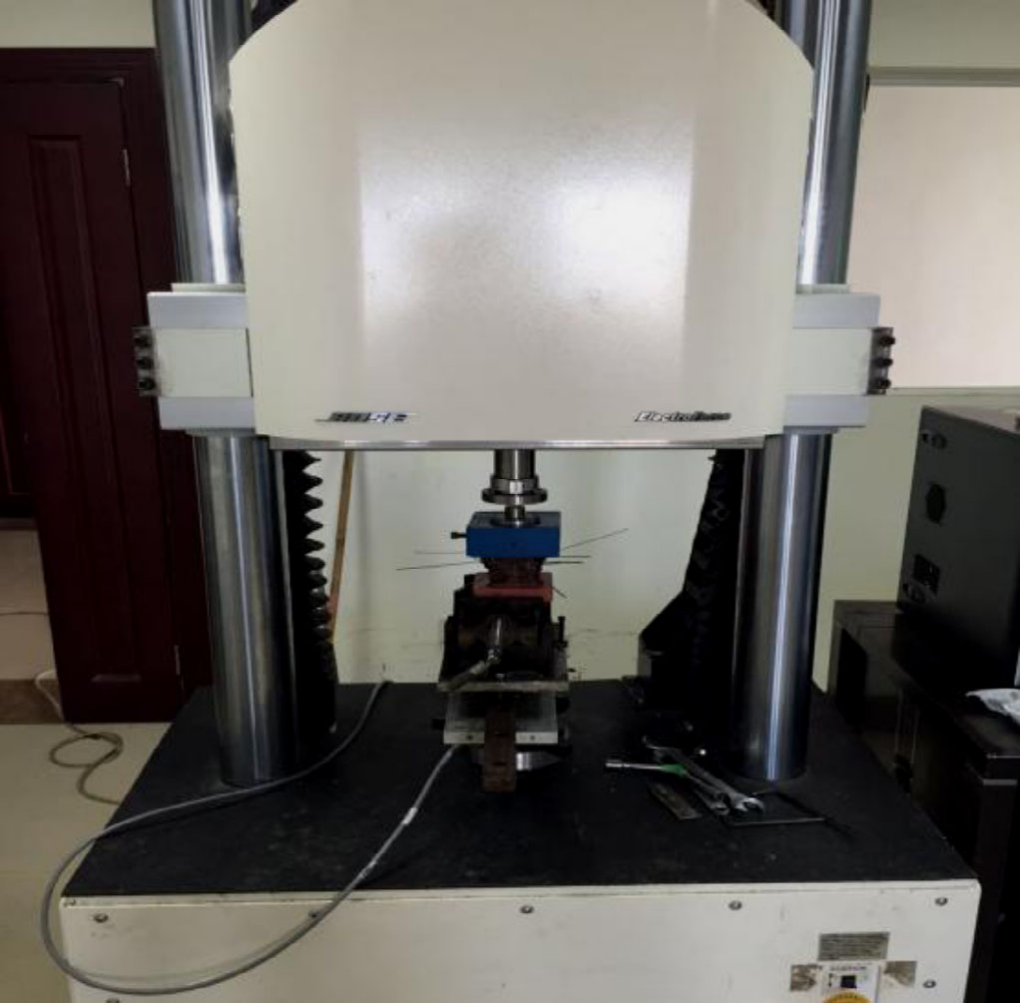
The prepared specimen was fixed on the 3D‐spine activity‐testing machine, according to the center of gravity of the skull loaded with a 100‐N weight to simulate the head weight and neck‐extensor muscle group. Through the 3D‐spine activity‐testing machine, we set the 1.5‐Nm torque to be slowly moving.

We selected a fresh cervical specimen of the body. Screws of 1 mm in diameter were screwed into the posterior foramen magnum, both sides of the foramen magnum, posterior nodules of atlas, bilateral transverse processes of atlas, and the spinous process of the axis and the bilateral transverse processes of the axis. This procedure required a total of nine screws. The skull base and the lower end of the specimen at the top of the C_3_ exposed subchondral bone into the plastic container, which exposed only the middle vertebrae (C_0_–C_3_) and cast a fixed substrate. The level of the upper and lower fixed platforms was not more than 0.1°; the foramen magnum was parallel to the horizontal plane to simulate the neutral position of the normal spine. We simulated the natural flexion and extension degrees of freedom of motion. The upper cervical spine specimens, after fatigue treatment, induced the cervical spine‐instability model, after which we measured the physiological flexion and extension degrees of freedom (Fig. 1).

### *Motion Capture*

**Figure 2:**
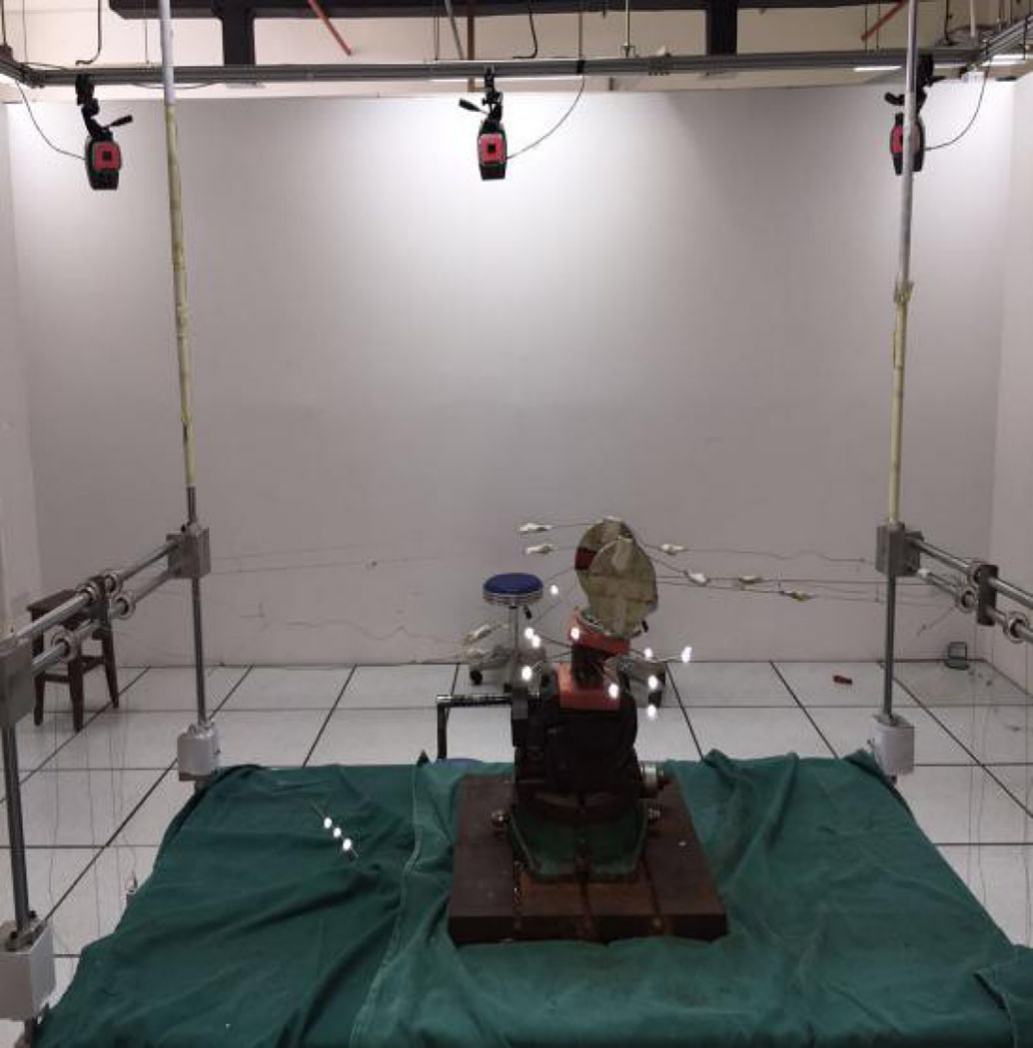
Ten 500‐Hz high‐speed infrared spot cameras fixed in the spine for measuring three‐dimensional activities were placed around the experimental machine, with a data cable being connected to the computer system to form a motion‐capture system. A 12‐gauge steel needle was placed into the C_0_‐C_2_ and was fixed, and each vertebral drilling was completed four times. After fixing the needle, the specially‐made marker light‐emitting point (marker point) was fixed on the free end of the needle.

Utilizing the motion‐capture system, the virtual probe was used to confirm the position of the screw in each vertebra and the four marker points of the vertebrae where the screw was located were tied to the screw position. Through this system, the dynamic tracking of the marker point fixed on the cervical spine during the simulation loading process was performed. The motion‐tracking data of the specimen were recorded completely for later use and analysis (Fig. 2).

### *Geometrical model*

**Figure 3:**
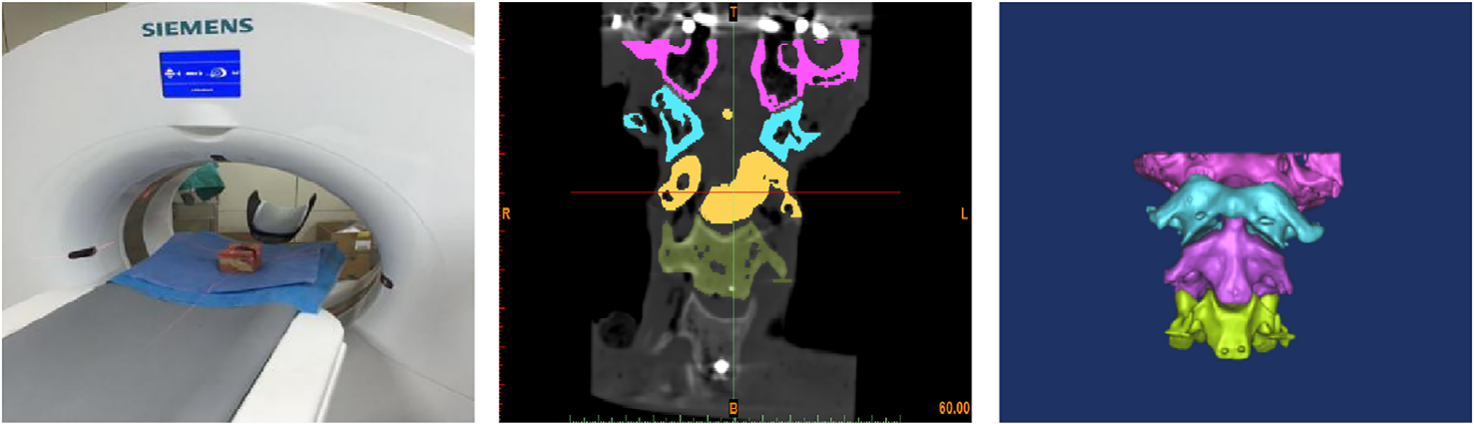
The CT machine was used to scan the upper cervical spine. Specifically, 219 tomograms were saved in the dicom format. In the segmentation module, the bone structure was selected by threshold selection in order to draw, erase, calculate three dimensions, smooth surfaces to perform other editing processes, and to obtain the corresponding position of the mark.

**Figure 4:**
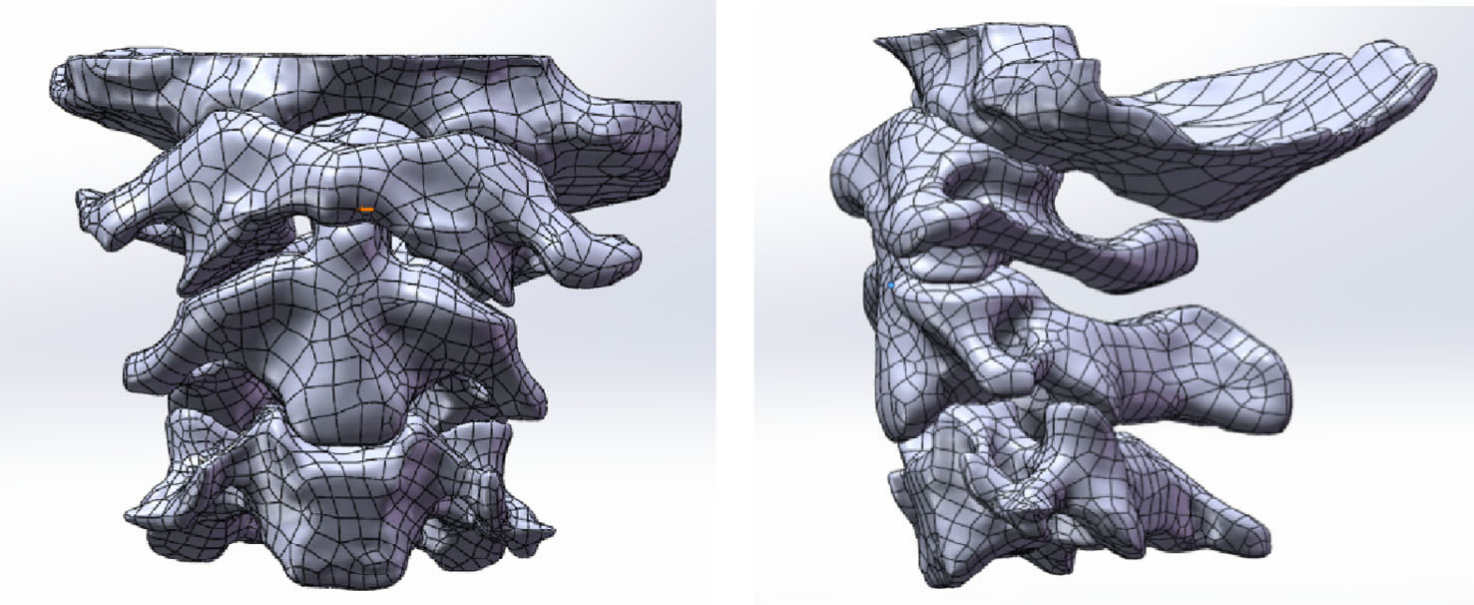
The point‐cloud data generated by the Mimics software was imported into the Solid Works of the 3D modeling software. Each file was saved as an igs‐format file.

**Figure 5:**
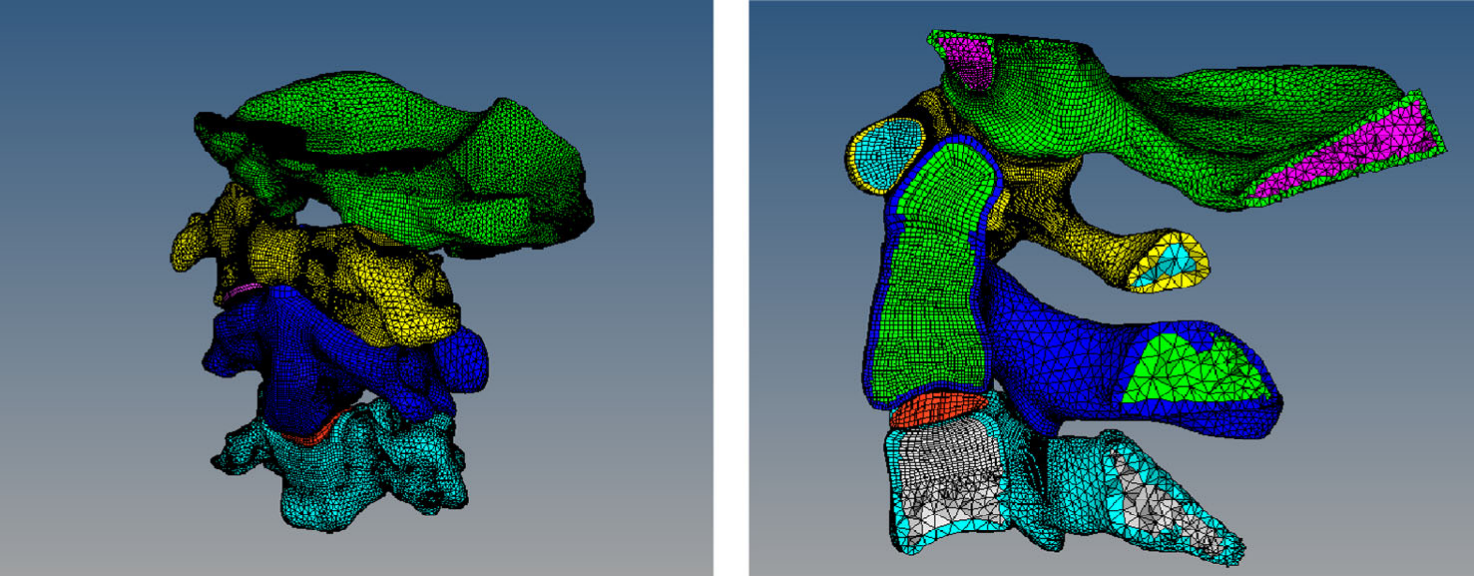
The solid‐map function was used to divide the hexahedron cells (C3D8R) into the contacted part and the 3D‐tetramesh to the tetrahedron mesh (C3D4). Transverse ligaments and articular cartilage were identified using the method of drawing hexahedron cells.

Our geometrical model was established as follows:

1. The DICOM (Digital Imaging and Communications in Medicine) format CT axial images were imported and processed by using Mimics software.
2. We established the part of the occipital and third cervical spine *via* 3D geometric modeling of the vertebrae (Fig. 3).
3. The whole outer surface of the bone structure was obtained through reverse processing (Fig. 4).
4. The established model was cut and segmented by using hypermesh (Fig. 5).

### *Geometrical Model and Experimental Data‐matching*

**Figure 6:**
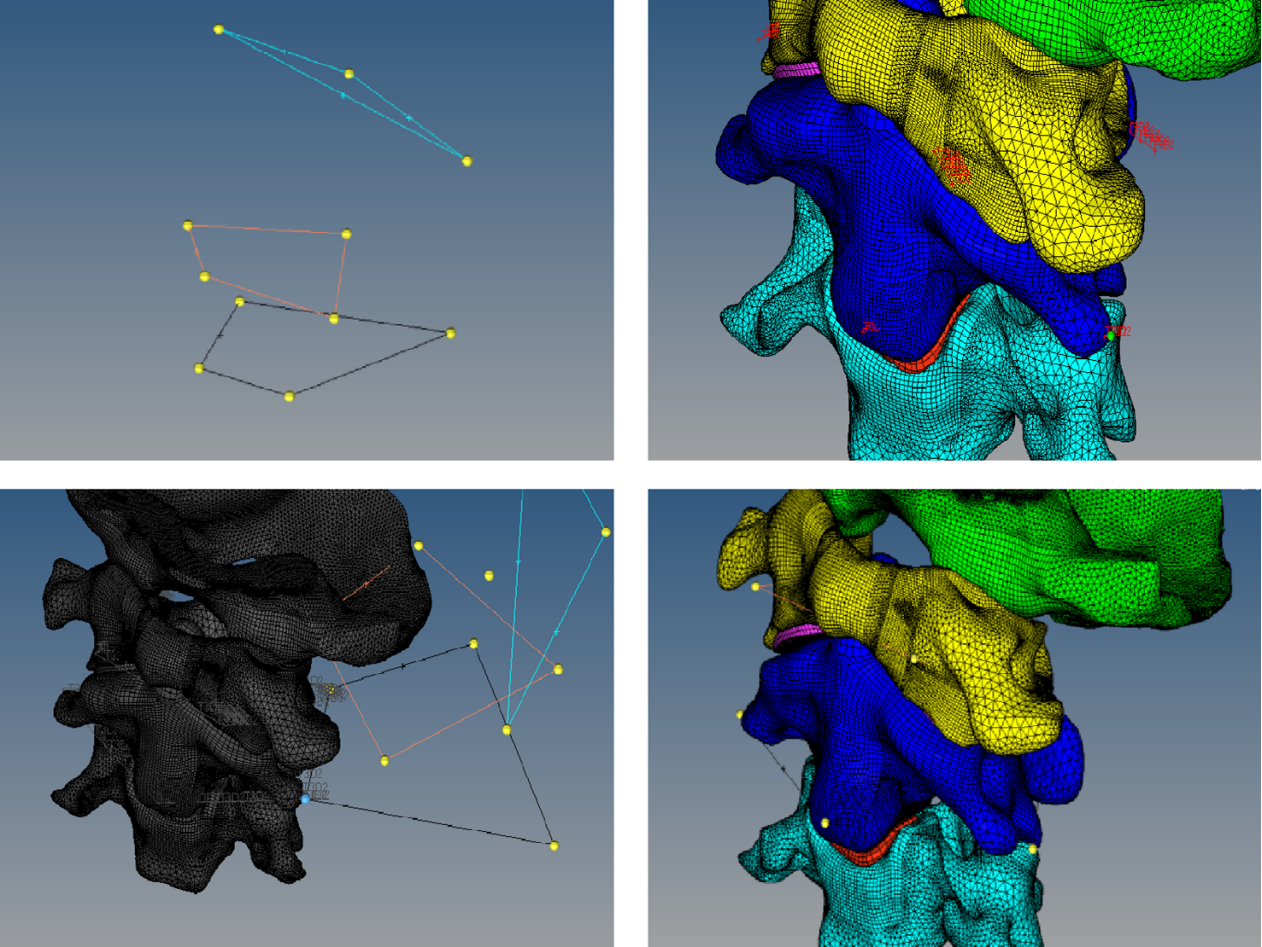
Taking three of the four virtual‐mark points of the central vertebrae of the geometric model as a triangle and regarding the triangle formed by the three points corresponding to the experimental model as two different spatial congruent or similar triangles, the use of the translation function after the former mark‐point shift to the latter corresponded to the marker point. Subsequently, we found the normal edge that contains the point, the normal axis, and the marker point. As the axis, the use of the spin function was used to identify the article side coincidence and the side of the line as the axis. This side of the edge of the other marker point for the axis will contain the marker point and the third mark of the edge of rotation overlap. Thus, the two triangles basically coincided, and the geometric model of the axis also moved to the experimental model origin.

The movement data of the flexion/extension activity point (approximately 1200 groups) collected from the *in vitro* specimen experiment were imported into the hypermesh software, and the neutral‐position data of the virtual‐mark points of each vertebrae were selected for registration. We established a simple geometric model and a virtual‐mark point corresponding to the experimental position (the specific mark‐point position can be imported from the mimics‐image model). In the experiment, the coordinate of the virtual‐mark point in the neutral position of each vertebrae was taken as the origin, and then the geometric model was moved to the position of the origin by the functions of rotation and translation of the software to perform the matching calibration in turn (Fig. 6).

### *Ligamentous Selection*

**Figure 7:**
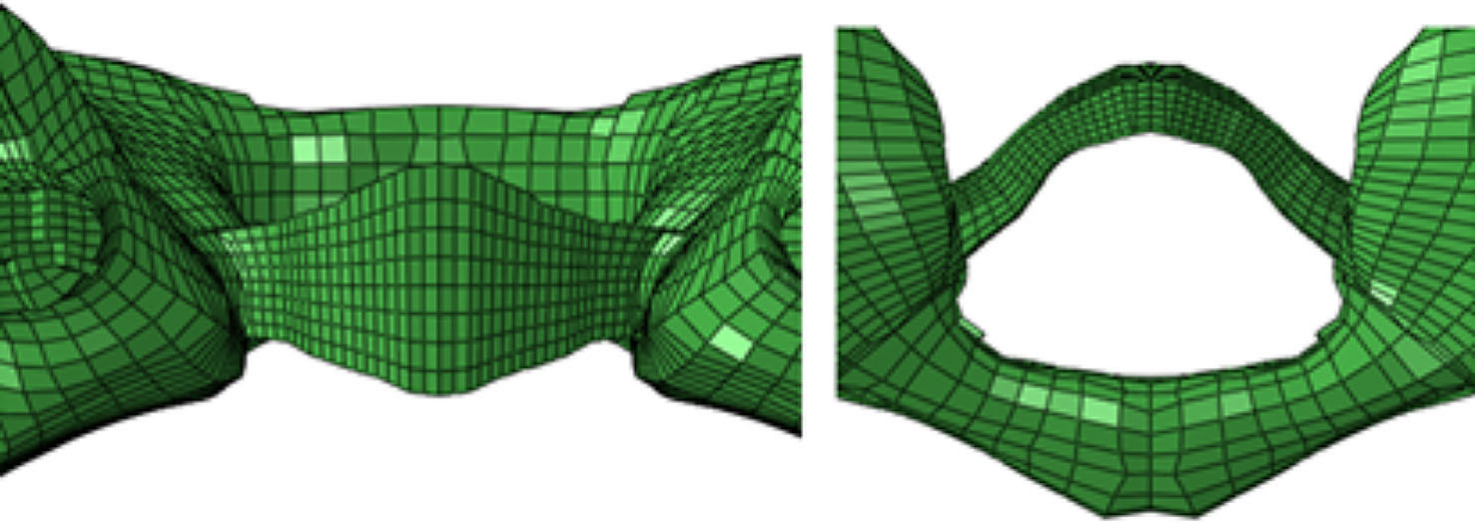
The transverse ligament‐mesh model was established based on the parameters of the transverse ligament of the cadavers^17^.

The nonlinear‐spring unit used in this study simulates the major ligaments of the transverse ligament in this region. We used reference‐related anatomical monographs^13^, ^14^, ^15^, ^16^ to determine the starting and ending point of ligaments and the establishment of ligaments simulated the spring unit, which included a total of 22 ligaments. The atlas transverse ligament and its front tooth formed joints, so the transverse ligament hexahedron unit was in a solid mode (Fig. 7).

### *Material Assignment*

Except for the transverse ligament, we used nonlinear parameters (i.e. nonlinear stress‐displacement curves). We used experimental data and related research literature^18^, ^19^, ^20^ to obtain the relevant material parameters (Table 1).

**Table 1:** Model material‐property settings

Parts	Elastic modulus (MPa)	Poisson's ratio	Cross‐sectional area (mm^2^)
Cortical bone	12,000	0.3	—
Cancellous bone	100	0.35	—
Cartilage	500	0.4	—
Intervertebral discs	20	0.45	—
Apical odontoid ligament			2.7
Tectorial membrane	20	0.45	1.2
Intertransverse ligament	50	0.45	13.1
Interspinal ligaments	28	0.45	13.1
Supraspinous ligament	28	0.45	13.1
Flavum ligament	50	0.45	50.1
Atlantoaxial collateral ligament	30	0.45	3.7
Occipital collateral ligament	30	0.45	4.1
Capsular ligament	20	0.45	46.6
Anterior longitudinal ligament	20	0.45	6.1
Posterior longitudinal ligament	70	0.45	5.4
Alar ligament	30	0.45	3.4
Anterior atlanto‐occipital membrane	20	0.45	2.8
Posterior atlanto‐occipital membrane	20	0.45	3.3

### *Contact Pairs*

Six pairs of contact pairs from C_0_ to C_3_ were established in this model. The Slave surface was the lower zygapophyseal‐joints surface of the upper vertebrae, and the Master surface was the upper zygapophyseal‐joints surface of the lower vertebrae. The joint surface was set as a sliding contact, the friction coefficient was set to 0.10, and the joint clearance was set to 0.01 mm.

### *Observational Factors*

There have been no clear imaging diagnostic criteria for upper cervical spine instability in the clinic. One study refers to the imaging criteria for cervical instability, which is that X‐rays show an angle of more than 11° between the vertebral bodies, or the horizontal displacement between the vertebral bodies exceeds 3.5 mm^4^. In addition, the selected stress‐observation indicator was the key ligament for maintaining the stability of the upper cervical spine.

The alar ligament extends from the tip of the odont to the inside of the occipital. The anterior longitudinal ligament (C_1,2_) originates from the edge of the occipital foramen and consists of three layers of juxtaposed fibers. They can play a certain role in the atlanto‐axial joint during the extension of the upper cervical spine.

The flavum ligament (C_1,2_) originates from the atlanto‐axial vertebra and consists of elastic fibers and collagen fibers. It can cause a significant restriction on the atlanto‐axial joint during the flexion of the upper cervical spine.

The posterior longitudinal ligament (C_2,3_) originates from the axis and consists of three layers of fibers. It can play a role in limiting the axial back‐shifting during the flexion of the upper cervical spine.

The apical odontoid ligament runs from the tip of the odontoid to the edge of the foramen magnum. The cruciate ligament is located behind the tooth process and consists of transverse and longitudinal fiber bundles. These ligaments can play a role in maintaining the stability of the atlanto‐axial joint during the flexion and extension of the upper cervical spine.

The atlanto‐occipital joint is mainly used during flexion and extension activities, while the atlantoaxial joint is mainly used for rotation activity, supplemented by flexion and extension activities. However, the relatively motorial angle of the atlanto‐occipital joint and the atlanto‐axial joint have never been clearly measured. The results of our present study may be used as an important reference for the degree of instability of the upper cervical spine.

## Results

### *Elements Contained in the Model*

**Figure 8:**
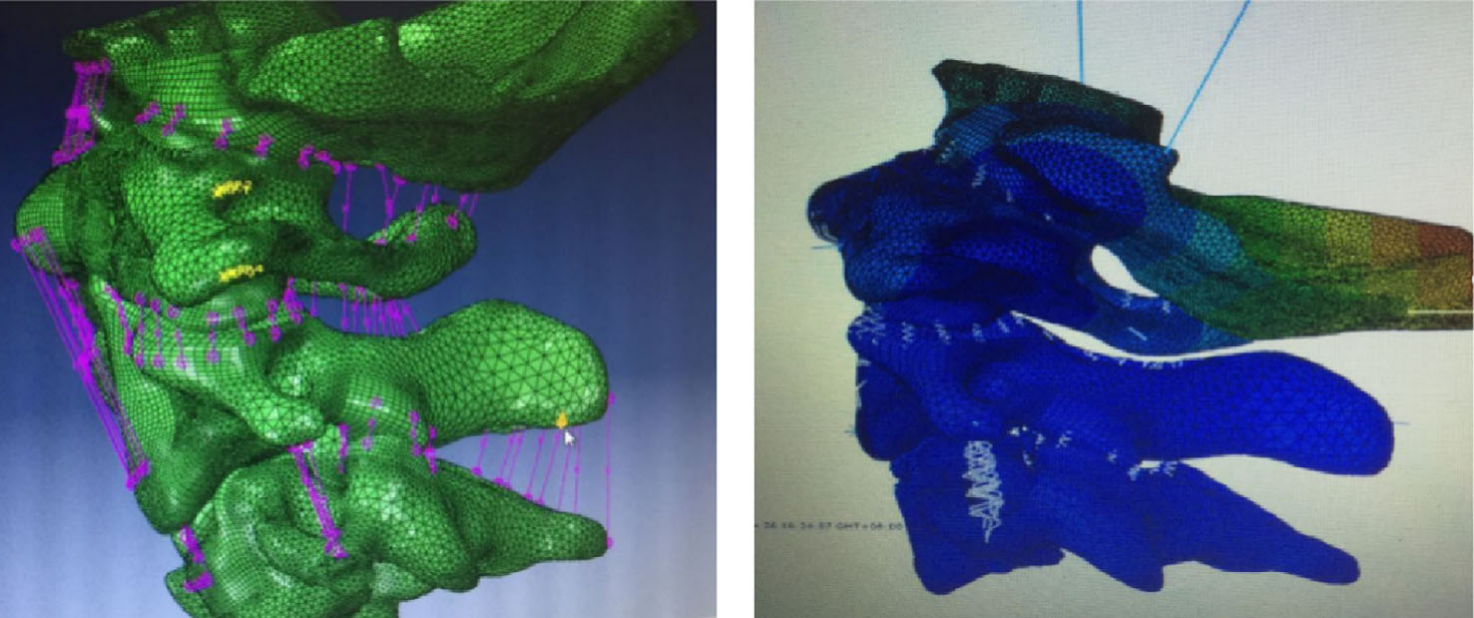
The unit types were divided into four types: C3D8, C3D8R, C3D8I, and C3D6. The tetrahedron element was 388560, the unit type was C3D4, the spring element was 183, the unit type was a spring, and the unit total was 627 000.

The model consists of an occipital bone, three vertebrae (C_1_–C_3_), 22 ligaments (1 apical odontoid ligament, 4 intertransverse ligaments, 2 interspinal ligaments, 1 supraspinous ligament, 2 flavum ligaments, 1 atlantoaxial collateral ligament, 1 occipital collateral ligament, 6 capsular ligaments, 1 anterior longitudinal ligament, 1 posterior longitudinal ligament, 1 alar ligament, and 1 cruciate ligament), a disc (C_2,3_), 6 pairs of articular cartilage (zygapophyseal joints surface), and a total of 238 257 hexahedron units (Fig. 8).

### *Ligamentous Stress Under Physiologic Conditions*

The stress of each major ligament during upper cervical spine flexion is shown in Table 2. The deformation of the ligament was most prominent in the flavum ligament, and the highest rigidity was the cruciate ligament. The stress per unit area was most obvious in the anterior longitudinal ligament and the flavum ligament. The ligament stress was the highest in the ligament.

**Table 2:** Stress of the ligament in flexion under physiological conditions

Ligaments	Length change (mm)	Rigidity (N/mm)	Stress (N)	Pressure (MPa)
Alar ligament	0.0708	25.32	1.792656	0.174044272
Ligamenta flavum (C_1,2_)	3.9	11.6	45.24	0.902994012
Anterior longitudinal ligament (C_1,2_)	1.957	24	46.968	7.699672131
Posterior longitudinal ligament (C_2,3_)	0.0559	26.34	1.472406	0.272667778
Apical odontoid ligament	0.915	28.6	26.169	10.4676
Cruciate ligament (Longitudinal)	0.409	38	15.542	2.285588235

The stress of each major ligament during the extension of the upper cervical spine is shown in Table 3. The deformation of the ligament was most prominent in the anterior longitudinal ligament of the atlantoaxial axis. The stress per unit area was most obvious in the dentate ligament and the anterior longitudinal ligament of the atlantoaxial ligament, and the posterior longitudinal ligament was almost unstressed.

**Table 3:** Stress of the ligament in extension under physiological conditions

Ligaments	Length change (mm)	Rigidity (N/mm)	Stress (N)	Pressure (MPa)
Alar ligament	1.159	25.32	29.34588	2.849114563
Ligamenta flavum (C_1,2_)	1.89	11.6	21.924	0.43760479
Anterior longitudinal ligament (C_1,2_)	2.666	24	63.984	10.48918033
Posterior longitudinal ligament (C_2,3_)	0.0278	26.34	0.732252	0.135602222
Apical odontoid ligament	2.24345	28.6	64.16267	25.665068
Cruciate ligament (longitudinal)	0.4153	38	15.7814	2.320794118

### *Relative Motorial Angle Under Physiologic Conditions*

During upper cervical spine flexion, the relative motorial angle of the atlanto‐occipital joint was 3.49°, and that of the atlanto‐axial joint was 8.84°. During upper cervical spine extension, the relative motorial angle of the atlanto‐occipital joint was 11.16°, and that of the atlanto‐axial joint was 14.20°. Under this condition, the relative motorial angle of the atlanto‐axial joint was more than that of the atlanto‐occipital joint, and the extension angles of the atlanto‐axial joint and the atlanto‐occipital joint were more than their flexion angles.

### *Ligamentous Stress Under Instability Conditions*

The stress of each major ligament during the upper cervical spine flexion is shown in Table 4. The ligament deformation was most prominent in the flavum ligament, and the highest rigidity was in the cruciate ligament. The stress per unit area was most obvious in the anterior longitudinal ligament and the flavum ligament. The ligament stress was the highest in the anterior longitudinal ligament. The stress of each major ligament during the extension of the upper cervical spine is shown in Table 5. The deformation of the ligament was most prominent in the anterior longitudinal ligament of the atlanto‐axial joint. The stress per unit area was most obvious in the talus ligament and the anterior longitudinal ligament of the atlanto‐axial ligament, and the posterior longitudinal ligament was almost unstressed.

**Table 4:** Stress of the ligament in flexion under instability conditions

Ligaments	Length change (mm)	Rigidity (N/mm)	Stress (N)	Pressure (MPa)
Alar ligament	0.2018	25.32	5.109576	0.49607534
Ligamenta flavum (C_1,2_)	5.227	11.6	60.6332	1.210243513
Anterior longitudinal ligament (C_1,2_)	2.566	24	61.584	10.0957377
Posterior longitudinal ligament (C_2,3_)	0.056	26.34	1.47504	0.273155556
Apical odontoid ligament	0.583	28.6	16.6738	6.66952
Cruciate ligament (longitudinal)	0.777	38	29.526	4.342058824

**Table 5:** Stress of the ligament in extension under instability conditions

Ligaments	Length change (mm)	Rigidity (N/mm)	Stress (N)	Pressure (MPa)
Alar ligament	3.303	25.32	88.035	8.12146443
Ligamenta flavum (C_1,2_)	2.53	11.6	29.35	0.58546877
Anterior longitudinal ligament (C_1,2_)	3.496	24	83.89	13.7522021
Posterior longitudinal ligament (C_2,3_)	0.028	26.34	0.7348	0.13554872
Apical odontoid ligament	1.429	28.6	40.87	16.351397
Cruciate ligament (longitudinal)	0.7889	38	29.97	4.40887307

### *Relative Motorial Angle Under Instability Conditions*

During upper cervical spine flexion, the relative motorial angle of the atlanto‐occipital joint was 5.51°, and that of the atlanto‐axial joint was 13.70°. During the upper cervical spine extension, the relative motorial angle of the atlanto‐occipital joint was 12.96°, and that of the atlanto‐axial joint was 17.20°.

Under this condition, the relative movement angle of the atlanto‐axial joint was more than that of the atlanto‐occipital joint, and the extension angles of the atlanto‐axial joint and the atlanto‐occipital joint were more than their flexion angles. After instability, in the direction of flexion and extension, the relative movement angle of the atlanto‐axial joint was significantly increased, while the atlanto‐occipital joint was not obvious.

### *Validation of the 3D‐dynamic Finite Element Model*

To validate the finite element model, we compared the strain values of specimen experiments with those of the upper cervical spine finite element model at each corresponding point by linear regression analysis. The loading and boundary conditions of the finite element model were the same as those of the specimen experiments. The regression equation and correlation coefficient were obtained as follows: *y* = 1.348*x* − 0.723, *R* ^2^ = 0.891. The *x*‐axis represents the finite element simulated equilibrium strains, and the *y*‐axis represents the strain values in the biomechanical experiment. The *R* ^2^ represents the correlation coefficient of the regression equation, which indicated that the finite‐element‐analysis results had a correlation with the experimental results.

### *Related Literature Research Verification*

Goel *et al*.^21^ used a 0.3‐Nm and Panjabi *et al*.^22^, ^23^ used a 1.5‐Nm load cadaver to test data. Initially, Goel *et al*. supposed that the 0.3‐Nm load on the C_0_ could lead to the movement of the upper cervical spine under physiological conditions. Comparing the relative movement angles between the atlanto‐occipital and atlanto‐axial bones calculated under two kinds of physiological loads with the results of the carcass test, the motion results of the model were all within the range of cadaver‐test data (Table 6).

**Table 6:** Comprehensive perspective verification

Movements	Segment	Literature angle data	Final model
Flexion	C_0_‐C_1_	1.9–11.4	3.49
	C_1_‐C_2_	1–8.8	8.84
Extension	C_0_‐C_1_	10.8–17.2	11.16
	C_1_‐C_2_	6.0–16.0	14.20

### *Verification of* in vitro *Specimen‐point Trajectory Data*

We calculated the angle data of *in vitro* specimen‐point trajectory data under physiological conditions, for which the atlanto‐occipital joint flexion was 3.82° and extension was 10.4°; atlanto‐axial joint flexion was 8.01° and extension was 14.89°. In the instability condition, the atlanto‐occipital joint flexion was 4.91° and extension was 12.55°; atlanto‐axial joint flexion was 14.08° and extension was 16.41°. The data were imported into SPSS 18.0 for statistical analysis, for which *P* > 0.05 indicated no significant difference. The results showed that the dynamic 3D model of upper cervical spine instability had a sufficient agreement with the *in vitro* specimen‐trajectory data. Accordingly, this model can truly and fully reflect the relevant biomechanical characteristics of the occipital–atlanto‐axial complex^24^, ^25^.

## Discussion

Upper cervical spine stability mainly depends on the stability of the atlanto‐occipital and the atlanto‐axial joint. However, due to the particularity of the anatomic structure of the upper cervical spine, this standard cannot be fully applied. Imaging‐based diagnosis of upper cervical spine instability has been challenging. Current widespread medical‐imaging methods cannot accurately capture and describe the entire disease process and dynamic characteristics of upper cervical spine instability. Applying this technology, however, could show the stress and deformation of the internal structure of the spine. Furthermore, such results have been displayed in an intuitive form^26^. Patients with upper cervical spine instability, especially during the symptoms period, can effectively avoid iatrogenic injury caused by dynamic‐position examination of the cervical spine by using these medical imaging methods^27^.

### *Mechanical Characteristics of Ligaments*

The results of the current study show that the alar ligament mainly bears the tension during upper cervical spine extension. Its deformation and stress were greatly increased after the instability of the upper cervical spine. The alar ligament was in a state of high stress, from 2.85 to 8.12 MPa, which indicated that the alar ligament played an important role in maintaining the stability of the atlanto‐axial joint during extension. Due to its attachment to the tip of the odontoid process, this will inevitably lead to deviation of the odontoid process, which causes atlanto‐axial instability. In contrast, in flexion, regardless of instability, the deformation and stress value of the alar ligament were not high. This indicates that the binding stress of the alar ligament to the atlanto‐axial joint was weaker in the flexion position.

There was high stress in the apical odontoid ligament during flexion or extension, but its stress and deformation were decreased after induction of instability. This result may be due to the slippage of the vertebrae during the flexion and extension of the unstable cervical spine and the distance between the tip of the odontoid process and the foramen magnum being shortened, resulting in the decrease of its stress. This also partly explains, after the instability, the influence of the position change between each vertebrae on the ligament stress.

Through data analysis, cruciate ligaments could be found with greater rigidity. The deformation and stress produced by the cruciate ligament changes little during flexion and extension. That is, the cruciate ligament had a certain constraint effect on the flexion and extension of the atlantoaxial joint, and the change of deformation and stress caused by instability was not obvious.

The anterior longitudinal ligament of the atlanto‐axial joint had a certain binding stress in the flexion and extension direction of the upper cervical spine, but the restraint effect was more obvious in the extension, especially after instability. The flavum ligament was the ligament connecting the laminae arcus vertebrae, which had the effect of limiting the vertebrae excessive flexion. It was not difficult to see from the experimental data that the stress and deformation were most obvious in the flexion position.

### *Articular Surface Changes in Relative Motorial Angle*

After instability, the movement angle of atlanto‐axial joint increased significantly during flexion, but the atlanto‐occipital joint was not obvious. During extension, the movement angle of the atlanto‐axial joint and the atlanto‐occipital joint were not obvious. Combined with the stress analysis of the ligament, it was considered that this may be due to the number of the limitation‐extension ligaments being higher and that its rigidity was greater. Therefore, when the cervical spine extends, the ligament produces a smaller variable shape variable, such that the change in angle was not obvious. In contrast, in the direction of flexion, the flavum ligament was mainly constraining the vertebrae, but its rigidity was low. Hence, the stress in the direction of flexion will produce greater deformation, resulting in a significant increase in the movement angle of the atlanto‐axial joint. This result also shows that frequent or prolonged flexion activities were more likely to cause damage to the upper cervical spine, leading to instability of the upper cervical spine. This can also partly explain why the large number of “phubbers” in our society are more likely to suffer from cervical spondylosis^28^, ^29^.

### *Limitations of the Current Study*

At present, there were few studies on 3D dynamic‐finite‐element models, and the existing theory remains insufficient. Therefore, there were still be many shortcomings in this type of research that will require improvements in the future. In addition, there are many types of instability of the upper cervical spine^30^. A model based on individual specimens does not fully explain the pathogenesis of upper cervical spine instability. It has certain limitations and needs more dynamic model data for further improvements. Finally, the human body is an active entity. The activity of the upper cervical spine contains muscles and nerves, in addition to joint ligaments. However, there have been few reports on data of muscles, blood vessels, and nerve excisions of the cervical spine. Therefore, the instability process of true cervical spine dynamics cannot be completely simulated, and the influence of vascular flow velocity on the neck cannot be evaluated^31^, ^32^.

## References

[1] Goel A , Kaswa A , Shah A . Role of atlantoaxial and subaxial spinal instability in pathogenesis of spinal ‘degeneration’ related cervical kyphosis. World Neurosurg, 2017, 101: 702–709. 28254542 10.1016/j.wneu.2017.02.063

[2] Meyer D , Kretschmer T , Börm W . Translaminar screws of the axis—an alternative technique for rigid screw fixation in upper cervical spine instability. Neurosurg Rev, 2012, 35: 255–261. 22086067 10.1007/s10143-011-0358-x

[3] Brolin K , Halldin P . Development of a finite element model of the upper cervical spine and a parameter study of ligament characteristics. Spine (Phila Pa 1976), 2004, 29: 376–385. 15094533 10.1097/01.brs.0000090820.99182.2d

[4] Dvorak J , Panjabi M , Gerber M , Wichmann W . CT‐functional diagnostics of the rotatory instability of upper cervical spine. 1. An experimental study on cadavers. Spine (Phila Pa 1976), 1987, 12: 197–205. 3589813 10.1097/00007632-198704000-00001

[5] Hutting N , Scholtenpeeters GG , Vijverman V , Martin DM , Arianne P . Diagnostic accuracy of upper cervical spine instability tests: a systematic review. Phys Ther, 2013, 93: 1686–1695. 23886844 10.2522/ptj.20130186

[6] Bess S , Harris JE , Turner AW , LaFage V , Smith JS , Shaffrey CI . The effect of posterior polyester tethers on the biomechanics of proximal junctional kyphosis: a finite element analysis. J Neurosurg Spine, 2017, 26: 1–9. 27611508 10.3171/2016.6.SPINE151477

[7] Mcafee PC , Bohlman HH , Han JS , Salvagno RT . Comparison of nuclear magnetic resonance imaging and computed tomography in the diagnosis of upper cervical spinal cord compression. Spine (Phila Pa 1976), 1986, 11: 295–304. 3750059 10.1097/00007632-198605000-00001

[8] Ng HW , Teo EC , Lee KK , Qiu TX . Finite element analysis of cervical spinal instability under physiologic loading. J Spinal Disord Tech, 2003, 16: 55–65. 12571486 10.1097/00024720-200302000-00010

[9] Mustafy T , El‐Rich M , Mesfar W , Moglo K . Investigation of impact loading rate effects on the ligamentous cervical spinal load‐partitioning using finite element model of functional spinal unit C2–C3. J Biomech, 2014, 47: 2891–2903. 25129167 10.1016/j.jbiomech.2014.07.016

[10] Mackiewicz A , Banach M , Denisiewicz A , Bedzinski R . Comparative studies of cervical spine anterior stabilization systems‐finite element analysis. Clin Biomech, 2015, 32: 72–79. 10.1016/j.clinbiomech.2015.11.016 26851563

[11] Mengoni M , Vasiljeva K , Jones AC , Tarsuslugil SM , Wilcox RK . Subject‐specific multi‐validation of a finite element model of ovine cervical functional spinal units. J Biomech, 2016, 49: 259–266. 26708919 10.1016/j.jbiomech.2015.12.005

[12] Pramudita JA , Kikuchi S , Minato I , Tanabe YJ . Effect of cervical spine alignment on neck injury risk during rear‐end impact‐numerical study using neck finite element model. Int J Crashworthiness, 2017, 22: 453–466.

[13] Agur AMR , Lee MJ . Grant's Atlas of Anatomy. 10th edn. Philadelphia: Lippincott Williams & Wilkins, 1999; 235–300.

[14] Xu N , Wei F , Liu X , Jiang L , Cai H , Li Z . Reconstruction of the upper cervical spine using a personalized 3D‐printed vertebral body in an adolescent with Ewing sarcoma. Spine (Phila Pa 1976), 2016, 41: 50–54. 10.1097/BRS.0000000000001179 26335676

[15] Richmond FJ , Bakker DA . Anatomical organization and sensory receptor content of soft tissues surrounding upper cervical vertebrae in the cat. J Neurophysiol, 1982, 48: 49–61. 6214617 10.1152/jn.1982.48.1.49

[16] Panjabi MM , Oxland TR , Parks EH . Quantitative anatomy of cervical spine ligaments. Part I. Upper cervical spine. J Spinal Disord, 1991, 4: 270–276. 1802157

[17] Sun J , Zhu QA , Lu HJ . The tensile strength of human transverse ligament of the atlas. Chin J Clin Anat, 1999, 17: 270–271.

[18] Kallemeyn N , Gandhi A, Kode S, Shivanna K , Smucker J , Grosland N . Validation of a C2–C7 cervical spine finite element model using specimen‐specific flexibility data. Med Eng Phys, 2010, 32: 482–489. 20392660 10.1016/j.medengphy.2010.03.001

[19] Claes LE , Heigele CA . Magnitudes of local stress and strain along bony surfaces predict the course and type of fracture healing. J Biomech, 1999, 32: 255–266. 10093025 10.1016/s0021-9290(98)00153-5

[20] Hu Y , Zhao HY , Hao MR . Biomechanical application of finite element method in upper cervical spine. Chin J Orthop Trauma, 2012, 25: 262–266. 22712384

[21] Goel VK , Clark CR , Gallaes K , Liu YK . Moment‐rotation relationships of the ligamentous occipito‐atlanto‐axial complex. J Biomech, 1988, 21: 673–680. 3170621 10.1016/0021-9290(88)90204-7

[22] Panjabi MM , Dvorak J , Crisco J , Oda T , Wang P , Grob D . Effects of alar ligament transaction on upper cervical spine rotation. J Orthop Res, 1991, 9: 584–593. 2045985 10.1002/jor.1100090415

[23] Kopperdahl DL , Moan EF , Keaveny TM . Quantitative computed tomograPhy estimates of the mechanical properties of human vertebral trabeeular bone. Orthop Res, 2002, 20: 801–805. 10.1016/S0736-0266(01)00185-1 12168670

[24] Viceconti M , Olsen S , Nolte LP , Burton K . Extracting clinically relevant data from finite element simulations. Clin Biomech, 2005, 20: 451–454. 10.1016/j.clinbiomech.2005.01.010 15836931

[25] Viceconti M , Bellingeri L , Cristofolini L , Toni A . A comparative study on different methods of automatic mesh generation of human femurs. Med Eng Phys, 1998, 20: 1–10. 9664280 10.1016/s1350-4533(97)00049-0

[26] Hou B , Wang Y , Shen YH . Mechanical analysis of knee dynamic finite element model. J Clin Rehabili Tis Eng Res, 2013, 22: 3998–4004.

[27] Kim YH , Khuyagbaatar B , Kim K . Recent advances in finite element modeling of the human cervical spine. J Mech Sci Technol, 2018, 32: 1–10.

[28] Fice JB , Cronin DS . Investigation of whiplash injuries in the upper cervical spine using a detailed neck model. J Biomech, 2012, 45: 1098–1102. 22284991 10.1016/j.jbiomech.2012.01.016

[29] Ying J , Teng H , Qian Y , Hu Y , Wen T , Ruan D . Radiographic analysis of the correlation between ossification of the nuchal ligament and sagittal alignment and segmental stability of the cervical spine in patients with cervical spondylotic myelopathy. Acta Radiol, 2018, 60: 196–203. 29788751 10.1177/0284185118778866

[30] Kettler A , Hartwig E , Schultheiss M , Claes L , Wilke HJ . Mechanically simulated muscle forces strongly stabilize intact and injured upper cervical spine specimens. J Biomech, 2002, 35: 339–346. 11858809 10.1016/s0021-9290(01)00206-8

[31] Wang ZP , Zhang XG , Zhao WT , Zhao XY . Biomechanical research progress on finite element analysis in the treatment of spinal manipulation. J Med Biomech, 2017, 32: 293–298.

[32] Lasswell TL , Cronin DS , Medley JB , Rasoulinejadb P . Incorporating ligament laxity in a finite element model for the upper cervical spine. Spine J, 2017, 17: 1755–1764. 28673824 10.1016/j.spinee.2017.06.040

